# Habituation with apparatus and group testing improves assessment of fish preferences

**DOI:** 10.1111/jfb.16053

**Published:** 2025-01-04

**Authors:** Chiara Varracchio, Francesco Pio Paci, Cristiano Bertolucci, Giorgio Bertorelle, Tyrone Lucon‐Xiccato

**Affiliations:** ^1^ Department of Life Sciences and Biotechnology University of Ferrara Ferrara Italy; ^2^ Department of Sciences, Technologies and Society, University School for Advanced Studies IUSS Pavia Italy

**Keywords:** animal‐based indicators, choice test, fish cognition, welfare assessment

## Abstract

Preference tests are commonly used to assess fish behavior and cognition in several research fields. This study aimed to investigate how fish perform in a preference test involving extended habituation to the apparatus, which was expected to reduce stress. We contrasted the choice between a sector of the apparatus with natural vegetation, expected to be the preferred stimulus, and a barren sector. Initially, we demonstrated that guppies' preference for the sector with vegetation increased after a 5‐day habituation period (Experiment 1). Subsequent experiments systematically modified the testing paradigm to observe effects on the preference. Experiment 2 introduced a physical separation between sectors to facilitate discrete choices, Experiment 3 tested groups of fish, and Experiment 4 used wild guppies. Only the modification in Experiment 3 impacted preference scores: guppies tested in groups showed a higher preference for the vegetation stimulus and spent less time in the central, no‐choice sector of the testing apparatus. Overall, this study supports the importance of methodological details in preference tests and highlights the benefits of extended habituation and group testing. Researchers should consider these factors when designing experiments to evaluate cognitive abilities or behavioral preferences in fish. Tailoring testing paradigms to specific research goals can improve the reliability and comparability of results, contributing to a deeper understanding of fish behavior and welfare.

## 1 INTRODUCTION

Preference tests are research paradigms in which an animal is presented with a choice between two or more different stimuli. Based on the subject's spontaneous behavior, the experimenter determines whether one of the stimuli is preferred over the other. Although applied across virtually all animal groups (mammals: Fernandez et al., 2004; Harri et al., 2000; Enke et al., 2022; birds: Ross et al., 2013; Stevens et al., 2021; Lemaire, 2020; reptiles: Mehrkam & Dorey, 2014; Tetzlaff et al., 2018; Hoehfurtner et al., 2021; amphibians: Lucon‐Xiccato et al., 2018; Ramos & Ortiz‐Díez, 2021, Unger et al., 2020; invertebrates: Gatto & Carlesso, 2019; Bengochea et al., 2023; d'Ettorre et al., 2021), preference tests are particularly common in fish research.

Preference tests are frequently used in studies on social behavior (Cote et al., 2012; Hager & Helfman, 1991; Hiermes et al., 2015; Krause et al., 1999; Krause & Godin, 1994) and sexual selection (Amorim et al., 2013; Amundsen & Forsgren, 2001; Evans et al., 2004; Houde, 1987; Laubu et al., 2017; Noonan, 1983). In these contexts, the stimuli are conspecifics, such as two groups of social companions or two potential mates. The choices made by the focal subjects provide information about preferred social companions or more attractive mates. Another important application of preference tests is in cognitive research. Here, analysing the behavioral choices of fish can offer insights into the underlying cognitive mechanisms. For example, if a subject shows a significant preference for one stimulus over another, it indicates that it has the cognitive abilities necessary to discriminate between the two stimuli. Using this approach, preference tests have been employed to study discrimination abilities for visual stimuli such as colors (Avdesh et al., 2010; Bruzzone et al., 2020; Oliveira et al., 2015), shapes (Caioni et al., 2023; Gatto, Bruzzone et al., 2021; Oliveira et al., 2015), sizes (Oliveira et al., 2015), and objects with different orientations (Gatto, Santacà et al., 2021). Additionally, preference tests have been used to study olfactory discriminations (Coppock et al., 2013; Lucon‐Xiccato et al., 2024) and numerical abilities (Agrillo & Dadda, 2007; Gómez‐Laplaza & Gerlai, 2011; Lucon‐Xiccato et al., 2023). Recently, the interest in preference tests has grown due to their applications in animal welfare. By presenting animals with a choice between different maintenance environments, researchers can directly determine their preferred conditions, which are considered indicators of the best captive conditions to improve animals' welfare (DePasquale et al., 2020; Graham et al., 2018; Kistler et al., 2011; Varracchio et al., 2024).

Despite their simplicity, preference tests have several limitations. First, null results are often difficult to interpret. If a subject does not display a significant preference between the stimuli, this could be due to (1) an actual absence of preference, (2) insufficient underlying cognitive abilities, or (3) lack of motivation to make a choice (Rowe & Healy, 2014). For example, an animal may not show a mate preference if it is not motivated to mate under the specific (e.g., unfamiliar) testing conditions. The second major issue, which we addressed in this study, is that most choice paradigms rely on the responses of subjects in an unfamiliar, potentially stressful testing environment. Although this setting may motivate certain choices, such as preferring an environment with refuges (Lucon‐Xiccato et al., 2023) or a large group of conspecifics (Hager & Helfman, 1991), it might inhibit other choices, such as those related to reproduction. Furthermore, the stress caused by the unfamiliar environment could negatively impact the cognitive abilities necessary for discrimination (e.g., Larsson et al., 2002; Liu et al., 2017; Rivera et al., 2020).

In this study, we aimed to develop a paradigm for the analyses of relatively long‐term preferences in guppies (*Poecilia reticulata*). The guppy is one of the most frequently used species to study fish behavior and cognition (e.g., Budaev, 1997; Godin & Davis, 1995; Jeswiet & Godin, 2011; Swaney et al., 2001; Trompf & Brown, 2014). In our preference test, fish behavior was scored after 5 days of habituation, a duration chosen to allow the subjects sufficient time to familiarize with the testing environment, thereby reducing stress‐related influences on their choices. We contrasted a simple pair of stimuli expected to elicit a strong preference: an environment with natural vegetation (i.e., aquatic plants) versus a barren environment (DePasquale et al., 2019; Graham et al., 2018; Schroeder et al., 2014). Initially, we assayed subjects from a common domestic strain in a simple rectangular area with the two stimuli positioned at the short walls (Experiment 1). We predicted that the subjects' preference for the plant stimuli would be increased by the extended testing time. Because we detected only a moderate preference for the environment enriched with vegetation, we conducted a series of additional experiments to identify experimental conditions that would increase the power to detect preferences. These additional experiments involved altering the apparatus (Experiment 2), changing the number of subjects tested (Experiment 3), and varying the strain of the subjects (Experiment 4) while keeping the same stimulus (i.e., natural vegetation) to allow direct comparison with Experiment 1.

## 2 MATERIALS AND METHODS

### 2.1 Subjects and maintenance conditions

The studies involved 177 juvenile guppies belonging to two different strains. A subset of 68 guppies originated from a domestic strain commonly breed for ornamental purposes and often referred to as “snakeskin cobra green.” This domestic strain of guppies was used across three separate experiments as follows: Experiments 1: *N* = 24; Experiment 2: *N* = 24; and Experiment 3: *N* = 105. The laboratory population of these domestic guppies was established in 2012, with individuals purchased from a local shop. Since then, this population has been maintained within our facility.

The second guppy strain used was obtained in 2002 from a wild population in the lower Tacarigua River, Trinidad. This strain did not undergo any controlled breeding process and was used as the “wild‐type” strain in Experiment 4 (*N* = 24). The wild guppies were collected 2 months prior to the experiment from an artificial outdoor pool in Padova (Italy) where they are maintained in a natural‐like habitat rich in vegetation and free from human interference. The collection was carried out using hand‐nets, and the guppies were immediately transported to the laboratory in aerated buckets. In the laboratory, the wild guppies were maintained under the same conditions as the domestic guppies, as described below.

The guppies of the two strains were maintained under standardized laboratory conditions. They were housed in mixed‐sex groups in 400‐L plastic tanks. The water temperature was kept at 27 ± 1°C and the conductivity at 606.7 ± 60.18 μS/cm. Nitrite levels were maintained below 0.1 mg/L, and nitrate levels were kept below 50 mg/L. Each maintenance tank was equipped with an aerator (ACO‐5505, Hailea, CN) and mechanical, chemical, and biological filters to ensure optimal water conditions. Natural vegetation, including *Echinodorus bleheri* and *Taxiphyllum barbieri* plants, along with colorful glass marbles, provided environmental enrichment. Illumination was provided by 30 W fluorescent lamps (GRO‐LUX, Sylvania, IT), programmed for a 12‐h light cycle (7:00 a.m.–7:00 p.m.). Feeding protocols consisted of twice‐daily administrations of commercial dry food (Vipan Nature Tropical Flakes, Sera, DE) and *Artemia salina* nauplii (Ocean Nutrition, Essen, BE).

The subjects were juveniles of each strain and were randomly selected the day after birth to ensure that all subjects were experimentally naïve. To obtain these juveniles, newly born fish were collected daily from the maintenance tanks and isolated in groups in tanks identical to the experimental ones, but devoid of enrichment, for 3 days. When the subjects were 4 days old, they were moved individually (Experiments 1, 2, and 4) or in small groups (Experiment 3) to the experimental apparatus for the testing.

### 2.2 General description of the paradigm

**FIGURE 1:**
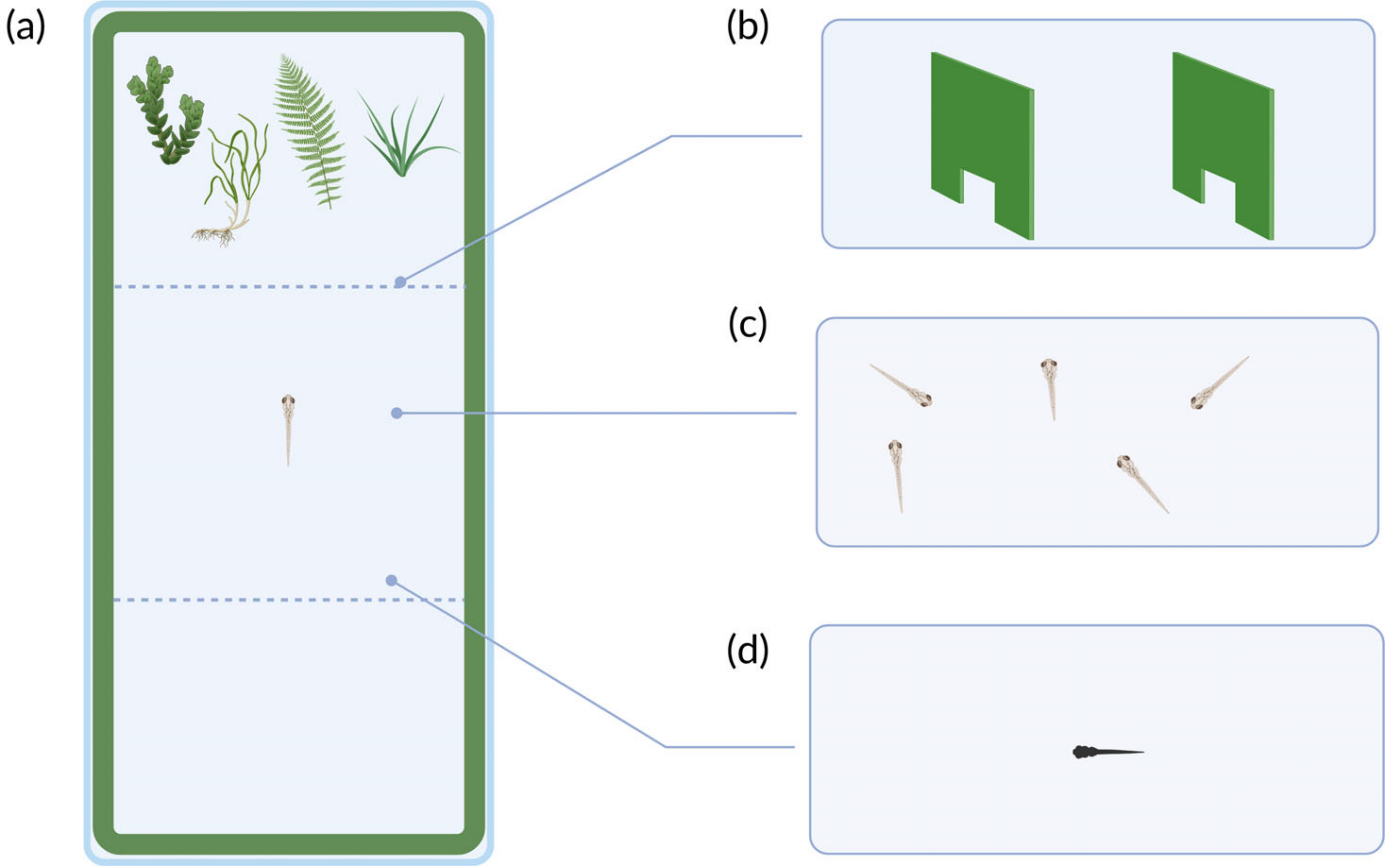
Schematic representation of apparatuses used in the study. (a) Version of the apparatus used in Experiment 1; the subjects (*N* = 24, domestic strain) were free to express their preference between the three “virtual” sectors of the tank. (b) Two perforated panels modified the apparatus of Experiment 2 to make the subjects' (*N* = 24, domestic strain) choice more discrete. (c) In Experiment 3, 21 groups of five individuals (*N* = 105 subjects, domestic strain) were tested in each apparatus. (d) In Experiment 4, a wild strain of guppies (*N* = 24 subjects) was assayed. The apparatus was as described in (a).

The apparatus was a 14 × 33 × 14‐cm tank filled with 10 cm of water (Figure 1). The tank was made of green plastic. We utilized 12 identical rectangular experimental apparatuses to test multiple subjects simultaneously. All the apparatuses were kept in the same room and maintained under the same conditions described for the maintenance of aquaria. A light‐emitting diode (LED) strip light source (20 W, 6500 K; TMR, distributed by ELCART, IT) was positioned above the center of each apparatus. A video camera (IMX179, ELP, Shenzhen Ailipu Technology Co., Ltd., CN; resolution: 3264 × 2448) was attached to the same support structure as the light source. The camera was used to record the behavior of the subjects during the testing by mean of a computer running the custom software “MultiViewer.”

The stimuli used for the preference experiment were plants belonging to four different species (*Limnophila heterophyla* “*sessilofora*,” *Egeria densa*, *Bacopa caroliniana*, and *Hygrophila polysperma*) purchased from a local store. To standardize the stimuli, the plants were trimmed down to obtain individual stems of 6 cm. Each stem was connected to a small weight consisting of a ceramic biofilter ring to prevent it from floating. In each trial, four stems of a single plant species were positioned at one end of the apparatus, within 10 cm from the short wall. The use of multiple species was intended to estimate a general preference for plants that was not strongly affected by the feature of a certain species. The opposite end of the apparatus was left empty to create a barren environment. In Experiment 1, a preliminary test demonstrated that the species of plant did not affect subjects' preference (*F*_3,20_ = 0.350, *p* = 0.789). The position of the enriched zone was alternated across the apparatuses and testing trials.

To commence the testing, a juvenile subject was randomly selected from a maintenance tank, collected with a net, and moved into the center of the apparatus. The subject was immediately fed *A. salina* nauplii, which was delivered using a Pasteur pipette in the central area of the apparatus. The subject was left in the apparatus for 5 days. During this period, the subject was fed *A. salina* twice per day. The observations necessary to collect the behavioral data were conducted by starting the camera as detailed in the schedule of the specific experiment. Each observation lasted 20 min. At the end of the experiment, all the subjects were released in a separate maintenance aquarium.

### 2.3 Detailed description of the experiments

Experiment 1 was used as a baseline consisting of the simplest version of the preference test (Figure 1a) in which we addressed the effect of extended testing time. The subjects (*N* = 24) were from the domestic strain, and the apparatus was as previously described. The behavioral preference of the subjects was observed twice. The first observation was conducted 30 min after the fish were moved into the apparatus. The second observation was performed after 5 days. The behavior of the subjects was scored offline exploiting the recordings. An experimenter played back the recordings on a computer monitor and activated a custom computer software (“Ciclic Timer”) consisting of multiple stopwatches controlled by the keyboard. The software allowed the experimenter to calculate the time spent in each of three sectors of the apparatus delimited by superimposing a transparent sheet with a reference to the monitor. The three sectors were delimited as follows: (1) sector with the stimuli (10 × 14 cm); (2) empty sector at the opposite end of the apparatus with respect to the stimuli (10 × 14 cm); and (3) central, no choice sector (13 × 14 cm; Figure 1a). The software was set to save an output each 2 min to analyse the behavior of the subject within observation. The time data were used to calculate the preference for the sector with the plants and the preference for the central, no‐choice sector.

In Experiment 2, the apparatus was slightly modified in an attempt to make the subject's (*N* = 24) choice more discrete. Previous studies have found that the apparatus used to score fish behavior may have a significant impact on the data collected (Dougherty & Shuker, 2015; Gatto et al., 2017; Gatto, Santacà et al., 2021; Gingins et al., 2018; Jones et al., 2023; Lucon‐Xiccato et al., 2017; Näslund et al., 2015). We reasoned that in Experiment 1 a subject could potentially see both the plant stimuli and the empty sector from each part of the apparatus. This may have reduced the subjects' motivation to choose between the two options. Therefore, in Experiment 2, two panels were placed in the apparatus to obtain three discrete sectors: one with the plant stimuli, a central no‐choice sector, and the barren sector (Figure 1b). The panels were perforated to permit the fish to move between sectors. However, the holes were relatively small (3 × 4 cm) and misaligned to prevent the fish from simultaneously seeing the two choice sectors. Twenty‐four fish were tested with this modified apparatus. The observations were conducted as described for Experiment 1, but only on the fifth day due to the strongest preference observed.

Experiment 3 replicated the testing conditions of Experiment 1 with a considerable variation: 21 groups of five individuals (total *N* = 105 subjects), instead individual subjects, were tested in each apparatus (Figure 1c). The guppy is a social species, usually observed in nature in groups of approximately 10–20 individuals (Seghers, 1974). Experiment 3 was intended to recreate a more natural setting that may improve the assessment of guppies' preferences. Due to the presence of multiple subjects in this experiment, the preference scores were measured by counting the number of subjects present in each of the three sectors. This scoring was performed in one frame per each block of 2 min of the recording. Based on the results of Experiment 1, the scoring was performed on the fifth day.

In Experiment 4, the modification with respect to Experiment 1 was the use of the wild strain of guppies (*N* = 24; Figure 1d). This adjustment was based on the evidence that the domestication process may alter the behavior of guppies (e.g., Gatto et al., 2018; Swaney et al., 2015). Using a wild strain, we expected to observe a more marked preference for the sector of the apparatus with the plant stimuli. Based on the results of Experiment 1, the scoring was performed on the fifth day.

### 2.4 Statistical analysis

All analyses were conducted in RStudio (version 2022.02.3, http://www.rstudio.com). The descriptive statistics in the Results section are presented as mean ± SD. The threshold for statistical significance was set at *p* = 0.05. As the first step of the analyses, we focused on the data regarding the central sector of the apparatus. This was used to compute a preference for the central sector, which was expected to provide indications of the efficiency of the test. For instance, a paradigm with lower preference for the central sector is likely to favor the estimation of the preference for the stimuli (Lucon‐Xiccato et al., 2017). In Experiments 1, 2, and 4, this preference was calculated as the proportion of time spent in the central sector (e.g., seconds spent in the central sector in each minute/60 s). The proportion of time spent in the central sector was fitted as dependent variable using linear mixed‐effects models testing for the effect of “block of minutes” to account for eventual variation in the preference (Krueger et al., 2020; Lucon‐Xiccato et al., 2016; Lucon‐Xiccato et al., 2017; Rosemberg et al., 2011). In Experiment 1, the day (e.g., day 1 vs. day 5) was also fitted as fixed effect. In Experiment 3, this analysis was conducted on the proportion of individuals in the central sector over the total number of subjects (e.g., 5). Notably, two subjects of Experiment 3 died on day 5, requiring to adjust the calculation accordingly. The dependent variables had normal distribution (Shapiro–Wilk normality test) except for Experiment 3, in which we applied a square root transformation to meet the assumptions of normality. In all these linear mixed‐effects models, subjects ID was fitted as random effect.

To analyse the preference for the stimuli, after confirming the normal distribution of data (Shapiro–Wilk normality test), we computed the proportion of time (Experiments 1, 2, and 4) or the proportion of subjects (Experiment 3) in the sector with the stimuli. These variables were first computed per each block of minute and analysed as described for the data of the central sector. Then, we compared the measure of preference against chance level (50%) using separate one‐sample *t*‐tests.

Last, to compare the observed preference for the central sector and the preference for the plant stimuli across experiments, we ran mixed‐effects models on the pooled data, fitting experiment as fixed effect. Post hoc analyses using Tukey's honestly significant difference (HSD) test were conducted to further analyse significant effects.

## 3 RESULTS

### 3.1 Experiment 1

**FIGURE 2:**
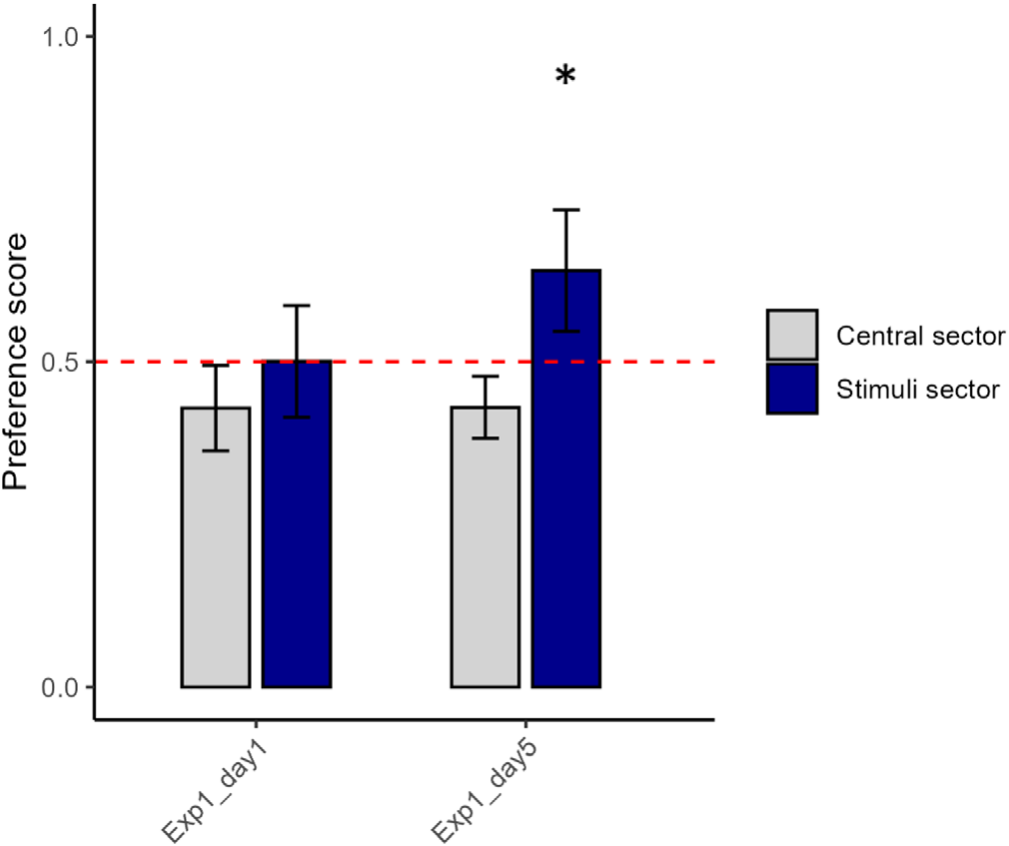
Effects of extended testing time on preference (Experiment 1). Proportion of time spent by the subjects (*N* = 24) in the central sector and in the sector with plants on the first and fifth days of testing. The panel displays the mean and 95% CI, whereas the dashed line represents the reference value of 50%, a threshold for interpreting the preference variable. *Indicates a significant deviation from that reference level.

On average, the subjects spent 257.65 ± 80.62 s in the central, no‐choice sector of the tank (Figure 2). The linear mixed‐effects model indicated a significant effect of minute (*F*_1453_ = 23.209, *p* < 0.0001) but no significant effect of day (*F*_1453_ = 0.004, *p* = 0.952; Figure 2) and no significant interaction between minute and day (*F*_1,453_ = 0.142, *p* = 0.706).

The preference for the sector of the apparatus with the plants indicated a significant effect of the day (*F*_1453_ = 22.152, *p* < 0.0001; Figure 2) but no significant effects of minute (*F*_1453_ = 2.061, *p* = 0.152) and no significant interaction between minute and day (*F*_1453_ = 1.927, *p* = 0.166). This suggested that the preference for the sector with the plants increased significantly from day 1 to day 5 (Figure 2). In agreement, we found that the preference for the sector with plants on day 1 was not significantly different from chance (50.04 ± 20.34%; one sample *t*‐test vs. 50% preference: *t*_23_ = 0.010, *p* = 0.991; Figure 2), whereas on day 5, the preference for the sector with plants was significantly greater than chance (64.00 ± 22.08%; one sample *t*‐test vs. 50% preference: *t*_23_ = 3.105, *p* = 0.004; Figure 2).

### 3.2 Experiment 2

**FIGURE 3:**
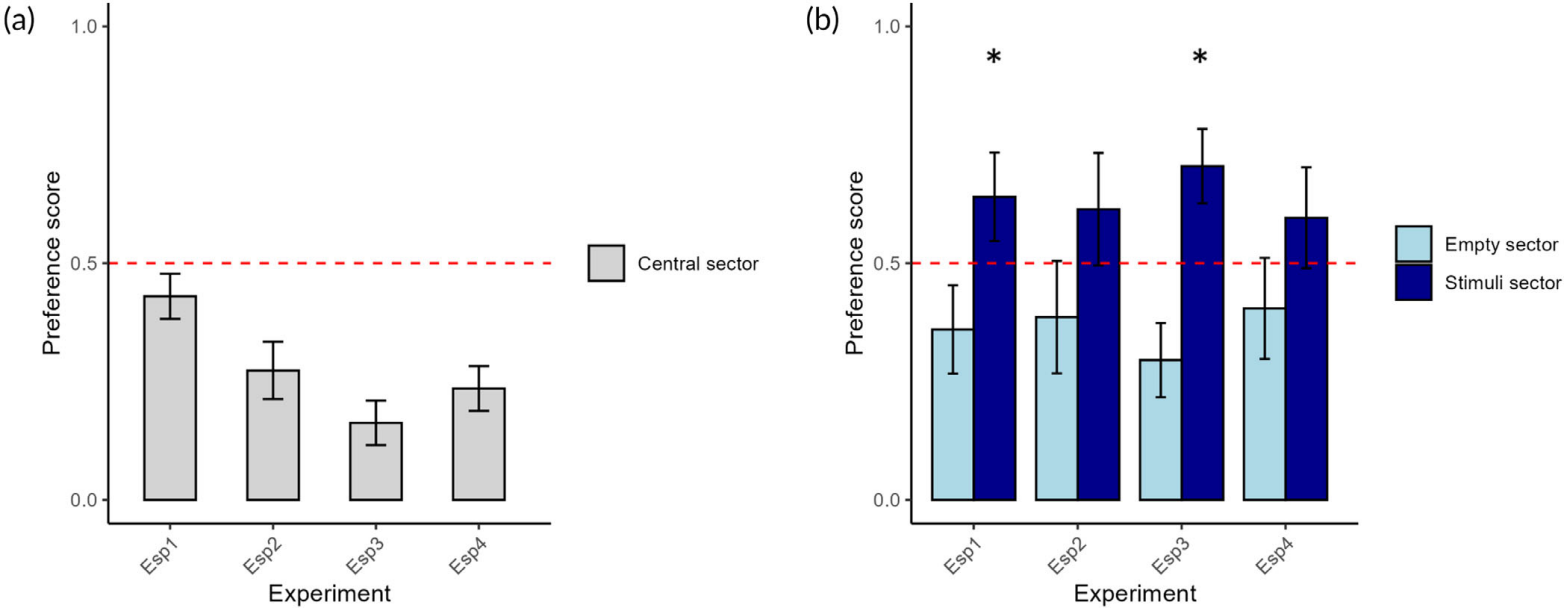
Comparison between experiments. Proportion of time spent in the central sector (a) and in the sector with plants versus the empty sector (b) on the fifth day of testing across all experiments (Experiment 1: *N* = 24; Experiment 2: *N* = 24; Experiment 3: *N* = 21 groups of five individuals; Experiment 4: *N* = 24). In both panels, means and 95% CI are displayed, whereas the dashed line represents the reference value of 50%, a threshold for interpreting the preference variable. *Indicate a significant deviation from that reference value.

The subjects spent 164 ± 85.90 s at the center of the tank (Figure 3a). The linear mixed‐effects model on this variable showed no effect of the minute (*F*_1215_ = 0.958, *p* = 0.329).

The preference for the sector of the apparatus with the plants was not affected by the minute (*F*_1213_ = 0.842, *p* = 0.360; Figure 3b). Considering the entire testing time, the preference for the sector with plants was not significantly greater than chance (61.40 ± 28.10%; one sample *t*‐test: *t*_23_ = 1.987, *p* = 0.058).

### 3.3 Experiment 3

The observed number of fish in the central, no‐choice sector of the apparatus was on average 7.48 ± 4.13 (Figure 3a). This variable was not affected by minute (*F*_1188_ = 2.858, *p* = 0.092).

The preference for the sector of the apparatus with plants was not significantly affected by the minute (*F*_1188_ = 1.016, *p* = 0.314). Moreover, the overall preference for the sector with the plants was significantly greater than chance (70.48 ± 17.22%; one sample *t*‐test: *t*_20_ = 5.451, *p* < 0.001; Figure 3b), indicating an interest of the fish for the natural environment.

### 3.4 Experiment 4

The wild strain of guppies spent 141.21 ± 67.05 s in the central sector of the apparatus (Figure 3a). This variable was not significantly affected by minute (*F*_1398_ = 2.371, *p* = 0.124).

The preference for the sector of the apparatus with the plants was not significantly affected by minute (*F*_1215_ = 0.064, *p* = 0.800). The overall preference was 59.57% ± 25.26%, which approached the statistical threshold for significant divergence from chance level (one sample *t*‐test: *t*_23_ = 1.855, *p* = 0.076; Figure 3b).

### 3.5 Comparison between the experiments

The analysis of the preference for the central, no‐choice sector indicated significant effects of the experiment (*F*_3899_ = 208.853, *p* < 0.0001; Figure 3a) and the minute (*F*_1899_ = 8.372, *p* = 0.004), along with a significant interaction experiment × minute (*F*_3899_ = 2.877, *p* = 0.035). The interaction was apparently due to the decreasing trend of Experiment 1. Post hoc analyses on the factor experiment indicated that lower preference for the central sector was obtained in Experiment 3 (vs. Experiment 1: *p* < 0.0001; vs. Experiment 2: *p* < 0.0001; vs. Experiment 4: *p* < 0.0001). Experiment 1 was characterized by the higher preference for the central sector (vs. Experiment 2: *p* < 0.0001; vs. Experiment 4: *p* < 0.0001), followed by Experiment 2 (vs. Experiment 3: *p* < 0.0001). There was no significant difference between Experiment 2 and Experiment 4 (*p* = 0.505).

The linear mixed‐effects model on preference for the sector with the plants on the fifth day indicated a significant effect of the experiment (*F*_3899_ = 6.783, *p* = 0.0002; Figure 3b). Post hoc analyses suggested that this main effect was due to the higher preference for the sector with plants displayed by the subjects in Experiment 3 (vs. Experiment 1: *p* = 0.013; vs. Experiment 2: *p* = 0.006; vs. Experiment 4: *p* < 0.001). There was no difference between Experiments 1 and 2 (*p* = 0.993), between Experiments 1 and 4 (*p* = 0.522), and between Experiments 2 and 4 (*p* = 0.692). The remaining terms in the model, the minute (*F*_1,899_ = 3.124, *p* = 0.077) and the interaction experiment × minute (*F*_3899_ = 0.422, *p* = 0.737) did not have a significant effect.

## 4 DISCUSSION

This study aimed to investigate how fish respond to a preference test conducted over a relatively long period of time. Although there are studies based on similarly long tests (DePasquale et al., 2020), most preference paradigms are conducted over a short period of time (e.g., Blaser & Goldsteinholm, 2012; Jones et al., 2019; Webster & Hart, 2004), potentially leading to behavioral alterations in response to the unfamiliar testing environment. The findings of Experiment 1 demonstrated that domestic guppies exhibited the expected preference for the stimuli presented (i.e., a sector of the choice apparatus with plants vs. an empty sector). However, this preference became evident only after 5 days of habituation to the testing apparatus. This finding highlights the importance of extended habituation periods in preference testing and similarly underscores the potential limitations of shorter testing durations in accurately assessing preferences. Although not assessed in our study, it is possible that a habituation longer than 5 days would better reflect the natural preferences of the tested animals.

Notably, the application of a long versus short testing period is dependent on the experimental design and goal. Only when the stress induced by the novel testing arena does not motivate the preference being assessed (Durrer et al., 2020; Fischer & Frommen, 2013; Frommen et al., 2009), longer testing periods may be preferable. Moreover, it cannot be excluded that a preference for a certain stimulus observed over a short period is qualitatively different from that observed over a long period. In our experiment, it is possible that initially, upon being introduced to the apparatus, subjects sought out plant stimuli as a refuge. However, over time, the sector with plants may have become a generally preferred living environment for the fish. Therefore, in studies aiming to assess animal welfare using preference tests (e.g., Dawkins, 1977; Hoehfurtner et al., 2021; DePasquale et al., 2020), longer testing periods appear to be more appropriate, whereas the contrary is true for studies looking at antipredator responses. The possibility of different underlying motivations for choosing the stimuli also underscores the need for caution when comparing results across studies with varying testing times. Furthermore, longer habituation time may improve discrimination performance between stimuli and are therefore more appropriate to assess the limit of the cognitive abilities of a certain species. For example, Gatto, Bruzzone et al. (2021) reported that guppies performed better in an operant conditioning task after residing in the testing chamber. Such effects could result from reduced stress, which is known to impair cognition (Li et al., 2008; Piato et al., 2011). Alternatively, subjects may allocate more attentional resources to stimulus choice when not on high alert in a novel environment.

Although the findings of Experiment 1 generally supported our hypothesis of better assessment of the preference with a longer habituation time, the average preference of guppies was modest (64%). This was unexpected given that the two choice sectors had a marked difference (presence vs. absence of vegetation; Sullivan et al., 2016). In the second part of the study, we aimed to enhance preference assessment by systematically modifying various aspects of the paradigm. Experiment 2, which introduced a physical separation between the two choice sectors, and Experiment 4, which involved using wild guppies, did not yield detectable improvements. However, Experiment 3, where individuals were tested in small groups, did result in a measurable increase in the preference, up to 70.5%. Another line of evidence for the improvement in Experiment 3 is the decrease in the time spent in the central, no‐choice sector of the apparatus. This indicated that the fish were more attracted to the choice sectors in Experiment 3, which is likely associated with greater possibility to assess their preference for the stimuli. Although the modest effect size observed warrants caution in interpretation, the results of Experiment 3 collectively indicate a positive effect of group testing in preference tests.

The results of Experiment 3 support earlier studies that the behavior of fish varies in group testing (e.g., Sommer‐Trembo et al., 2022; Jolles et al., 2017; Gentsch et al., 1983; Günzel et al., 2021). Various mechanisms may contribute to this modulation in the context of our experiment. Social fish often display the so‐called “social buffering effect,” wherein individuals experience reduced stress in the presence of conspecifics (Culbert et al., 2019; Pintos et al., 2024). Social buffering might therefore determine a more natural behavior in the fish and a more accurate assessment of their preference. Moreover, being in a group, each individual may decide which sector to choose based on the choices of their group mates. This phenomenon is expected to enhance overall discrimination performance (Laland & Williams, 1997; Ward et al., 2011). This is also true in the case of one or a few informed leaders that determine the choice of the entire group (Nakayama et al., 2016). For example, Bisazza et al. (2014) studied the preference for large social groups in guppies by testing either pairs of subjects simultaneously or single subjects. They found increased accuracy in the pair condition. Furthermore, they found that the accuracy of each pair matched the performance of the most skilled individual within the pair, suggesting that this individual acted as a leader and determined the overall performance of the pair. A similar effect might explain our results in Experiment 3.

Based on Experiment 3, we conclude that studies aiming to assess the maximum cognitive ability of a species or to evaluate general preferences, such as for welfare purposes, may benefit from testing fish in groups rather than individually. However, this approach also has drawbacks. For instance, it has been shown that shoals formed by fish of different sexes (Lucon‐Xiccato & Griggio, 2017; Schons et al., 2021) or with different personalities (Brown & Irving, 2014) display varying behaviors. This suggests that assessing the preferences of a group of subjects may not yield results that can be generalized to the entire species but rather apply only to the specific set of individuals tested. Therefore, the choice between individual and group testing greatly depends on the experimental goal and the nature of the species being studied.

## 5 CONCLUSIONS

Overall, our results suggest that the preference for “natural‐like” environments in guppies is significantly increased by extended habituation to the apparatus and group testing. This finding aligns with previous studies emphasizing the importance of methodological details in behavioral research in fish (Gatto et al., 2020; Gatto, Santacà et al., 2021; Jones et al., 2021; Lucon‐Xiccato et al., 2017; Prétôt et al., 2016; Swaney et al., 2024). Additionally, the results indicate that various versions of preference paradigms can be tailored to different research objectives. For instance, long testing time and group testing might be preferable to assess fish welfare and the upper limit of their discrimination capacities, at least when the choice is not motivated by the stress of the unfamiliar environment. Considering these methodological insights will likely contribute to improved standardization of research protocols and a greater understanding of fish behavior. This work should encourage studies exploring other factors that may influence preference tests, such as the distance between the two options (Swaney et al., 2024) and the shape of the testing environment (Jones et al., 2023).

## AUTHOR CONTRIBUTIONS

Conceptualization: Chiara Varracchio, Francesco Pio Paci, Cristiano Bertolucci, Giorgio Bertorelle, and Tyrone Lucon‐Xiccato. Methodology: Chiara Varracchio and Tyrone Lucon‐Xiccato. Formal analysis and investigation: Chiara Varracchio. Data curation: Chiara Varracchio and Francesco Pio Paci. Writing—original draft preparation: Chiara Varracchio and Tyrone Lucon‐Xiccato. Writing—review and editing: Chiara Varracchio, Francesco Pio Paci, Cristiano Bertolucci, Giorgio Bertorelle, and Tyrone Lucon‐Xiccato. Funding acquisition: Giorgio Bertorelle.

## FUNDING INFORMATION

This publication was produced while attending the PhD programme in PhD in Sustainable Development and Climate Change at the University School for Advanced Studies IUSS Pavia, Cycle XXXVIII, with the support of a scholarship financed by the Ministerial Decree number 351 of April 9, 2022, based on the NRRP‐funded by the European Union‐NextGenerationEU‐Mission 4 “Education and Research”, Component 1 “Enhancement of the offer of educational services: from nurseries to universities”‐Investment 4.1 “Extension of the number of research doctorates and innovative doctorates for public administration and cultural heritage”.

## CONFLICT OF INTEREST STATEMENT

The authors declare no conflict of interest.
